# Deciphering the molecular components of the *quorum sensing* system in the fungus *Ophiostoma piceae*

**DOI:** 10.1128/spectrum.00290-23

**Published:** 2023-10-05

**Authors:** Rodrigo Santos-Pascual, Iván Campoy, David Sanz Mata, María Jesús Martínez, Alicia Prieto, Jorge Barriuso

**Affiliations:** 1 Department of Microbial and Plant Biotechnology, Centro de Investigaciones Biológicas Margarita Salas, Consejo Superior de Investigaciones Científicas (CIB-CSIC), Madrid, Spain; Centro de Investigaciones Biologicas CSIC, Madrid, Spain

**Keywords:** *quorum sensing*, signaling, biotechnology, ecophysiology

## Abstract

*Quorum sensing* (QS) is a complex cell-cell communication mechanism that coordinates population-level behaviors in microbes. In eukaryotes, this phenomenon has been extensively described in the dimorphic yeast *Candida albicans* as its main QS molecule, the sesquiterpene alcohol farnesol, is responsible for various phenotypic (i.e., inhibition of yeast-to-hyphae transition, biofilm formation, and, hence, pathogenesis) and metabolic (i.e., induction of oxidative stress and apoptosis) changes. *Ophiostoma piceae* CECT 20416 is a dimorphic saprotrophic ascomycete with biotechnological interest that also produces farnesol as QS molecule, but in this case, the alcohol promotes the morphological transition to the mycelial form, biofilm formation, enzyme secretion, and melanin production. Here, we characterized the physiological response of *Ophiostoma piceae* to farnesol, the molecular components of the QS system of this fungus have been investigated using a “multiomics” approach that involved genomic, transcriptomic, and proteomic analyses. Some genes identified in this work are proposed as key factors in farnesol transport and signaling. We have also cataloged the genes undergoing major transcriptional changes triggered by the presence of the autoinducer, such as cell-wall remodeling, reactive oxygen species (ROS) protection, and melanin biosynthesis, using self-organizing maps. This analysis could be useful for applications in the forestry industry, for enzymes production, and for the valorization of residues. Furthermore, it might as well help to investigate the QS mechanisms of clinically relevant fungi phylogenetically related to *Ophiostoma*.

**IMPORTANCE:**

This manuscript presents a comprehensive study on the molecular mechanisms triggered by the *quorum sensing* (QS) molecule farnesol in the biotechnologically relevant fungus *Ophiostoma piceae*. We present for the first time, using a multiomics approach, an in-depth analysis of the QS response in a saprotroph fungus, detailing the molecular components involved in the response and their possible mechanisms of action. We think that these results are particularly relevant in the knowledge of the functioning of the QS in eukaryotes, as well as for the exploitation of these mechanisms.

## INTRODUCTION

*Quorum sensing* (QS) is a complex inter-cellular communication mechanism used by microorganisms to coordinate their population behaviors. This mechanism is mediated by small diffusible molecules, which accumulate throughout the microbial growth cycle until a threshold concentration is reached. Then, gene expression is altered and a coordinated response is triggered in the community (1). The first QS molecule (QSM) described was an acyl-homoserine lactone (AHL) from the bacterium *Aliivibrio fischeri* (2), and many different QSMs have been reported in bacteria thereafter. Further investigations revealed the existence of these cell-cell communication processes in eukaryotic microorganisms. In fungi, they modulate secondary metabolism, enzyme production, morphogenesis, or biofilm formation (3 – 11).

The QS mechanisms of the dimorphic opportunistic fungus *Candida albicans* are well studied, as they are essential to regulate processes directly related to pathogenesis, such as biofilm formation and morphological transition (12). Two molecules, farnesol (1-hydroxy-3,7,11-trimethyl-2,6,10-dodecatriene) and tyrosol, have been described to act as QS signals in this yeast (13, 14) although the effects of the sesquiterpene alcohol farnesol are better known. In *C. albicans*, farnesol triggers a complex regulation mechanism that blocks morphological transition and biofilm formation, acting as a negative regulator of filamentation. Among its effects, this QS molecule impacts the HOG MAPK pathway involved in osmosensing, cell wall biogenesis, and morphogenesis (15). In addition, induces farnesylation of the small GTPase-like protein Ras1 (4, 16) and, most importantly, inhibits the cyclic AMP-Protein kinase A (cAMP-PKA) signaling pathway. Repression of the adenylate cyclase gene (*cyr1*) by farnesol reduces the intracellular levels of cAMP, abolishing signal transduction to the protein kinase A cascade. As a consequence, there is no upregulation of the gene *efg1* encoding the Enhanced Filamentous Growth protein 1 (17), nor downregulation of *nrg1,* the major repressor of hyphal development. In addition, farnesol also acts at the protein level avoiding the degradation of Nrg1. This complex regulation leads to increased levels of Tup1, a DNA-binding transcriptional regulator that suppresses hyphal formation (18).

On the other hand, the crosstalk between microorganisms from different kingdoms of life that share the same habitat is known to occur routinely in nature (19, 20). As an example, *C. albicans* responds to 3-oxododecanoyl-l-homoserine lactone (3-oxo-C12-HSL), the QSM from the bacterium *P. aeruginosa,* which inhibits Cyr1 and, subsequently, yeast filamentation, in a dose-dependent manner. Similarly, farnesol represses the *Pseudomonas* QS system mediated by quinolones (21, 22).

The ascomycete *Ophiostoma piceae*, class Sordariomycetes, is a non-pathogenic dimorphic saprotrophic fungus responsible for the conifer sap-stain, which causes financial loses in the wood industry (23). Its ability to synthesize melanin—the staining agent—as well as to secrete several enzymes such as proteases, laccases, or lipases, makes *O. piceae* have a high biotechnological potential (24 – 26). In this fungus, the morphological transition from yeast to hyphae is also a major driver of several physiological abilities such as the secretion of enzymes (i.e., the versatile lipase OPE, which is the major esterase secreted and has broad substrate specificity) and the formation of biofilms by modulating cell surface hydrophobicity (27 – 29). These traits are key to enable adaptability and colonization, as seen in other *Ophiostoma* species (i.e., *O. ulmi* and *O. novo-ulmi*) (30). Factors like inoculum size, population density, temperature, nitrogen source, or QS signals have been described to induce the dimorphic switching in *Ophiostoma* species (31 – 33). The strain *O. piceae* CECT 20416 produces farnesol as QS molecule (27) which, unlike the situation in *C. albicans,* induces the yeast-to-hypha transition. Homologs to *cyr1* and *tup1* have been identified in this species (27), but little information is available on their transcriptional regulation and the components involved in the farnesol-mediated signaling pathway.

DNA and RNA sequencing technologies, together with the vast amount of genomic information in public repositories, make multiomics approaches powerful techniques for revealing the genetic response of organisms to different stimuli (34). In this work, we have combined genomics, transcriptomics, and proteomics to unravel the main molecular components involved in the QS response of *O. piceae* CECT 20416. Here, we present the first approach toward understanding farnesol signaling, with particular attention to the regulatory genes involved in dimorphism, the synthesis of secondary metabolites and relevant enzymes, and their time-dependent variations. These results can be extrapolated to phylogenetically related fungi with clinical and biotechnological implications.

## RESULTS

### Effect of culture conditions on the growth and morphology of *Ophiostoma piceae*

**Fig 1:**
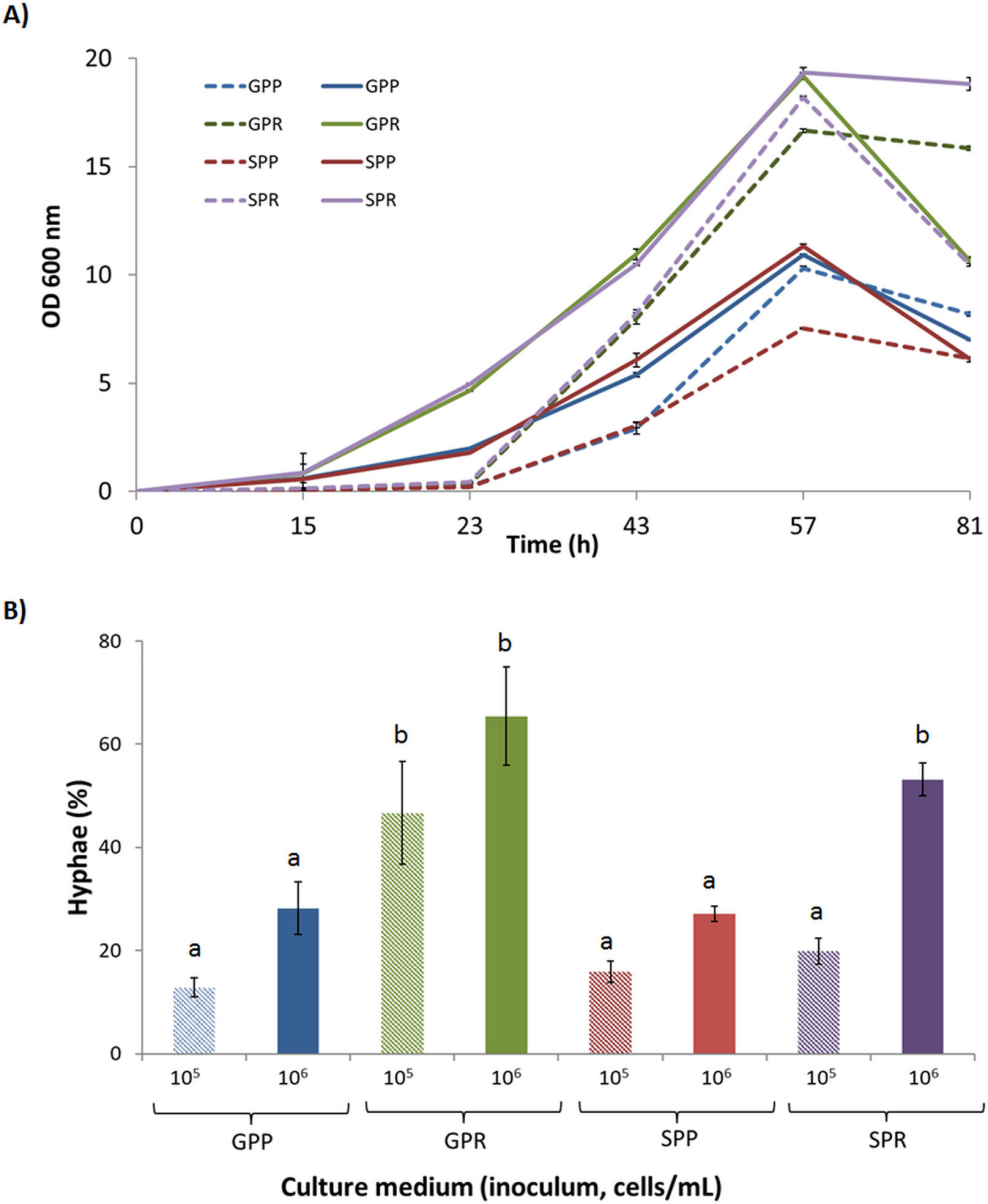
Determination of growth and morphology in *O. piceae* CECT 20416 cultures in phosphate medium with glucose (G) or sucrose (S) as carbon source and proline (P) or arginine (R) as nitrogen source. Cultures were inoculated at low (105 cells/mL, striped bars) and high cell density (106 cells/mL, solid bars). The samples were filtered through Miracloth to separate hyphae (retained) from yeast cells (flowthrough). (A) Optical density (OD 600 nm) of the yeast population was determined throughout the experiment. (B) Percentage of cells in the form of hyphae at 47 h, as observed under light microscope. Eighteen biological replicates were used both for control samples and for the cultures induced with farnesol. Groups of six biological replicates were pooled together to give three technical replicates that were individually analyzed. Error bars: standard error. Statistical significance of the results was evaluated by ANOVA and Tukey tests.

In order to evaluate the optimal culture conditions to elucidate the farnesol signaling response in *O. piceae*, various inoculum densities and carbon and nitrogen sources were tested in phosphate minimal medium. Growth correlated with inoculum size and the exponential phase began earlier in cultures with high inoculum size (10^6^ yeast/mL). Furthermore, cell growth was higher when arginine was used as the nitrogen source. The carbon sources tested, glucose or sucrose, had not significant effect on growth rates (Fig. 1A). Regarding cell morphology, the proportion of hyphae during the exponential growth phase (47 h) was significantly higher in cultures with glucose, high inoculum density, and arginine as the nitrogen source (Fig. 1B; Fig. S1). Cell counts under the microscope showed a proportion of hyphae around 10%–20% in the low inoculum density cultures with proline, between 30% and 50% in the high inoculum density cultures with proline, 50%–65% in the low inoculum density cultures with arginine, and 65%–70% in the high inoculum density cultures with arginine (Fig. 1B). The morphological transition from yeast to hyphae in the cultures was corroborated by the increase in both, the mycelium dry weight in the cultures with arginine (Fig. S1B), and the shape of the cells, determined by microscopy (Fig. S1).

### Effect of farnesol on the morphological transition and esterase activity

Since the lowest proportion of hyphae in *O. piceae* CECT 20416 was observed using GPP medium (Fig. 1B), this was selected to assess the inductive effect of farnesol on yeast-to-hyphal transition and extracellular enzyme (esterase/lipase) production. Cultures growing in GPP inoculated with a low inoculum concentration (10^5^ cells/mL) were induced with 100 µM farnesol 24 h after inoculation, keeping controls without induction (Fig. S2A). The highest presence of hyphae in the exponential growth phase (47 h) was observed in cultures induced with farnesol (42%) compared with the non-induced culture (8%) (Fig. S2B and C). Moreover, we determined the esterase activity in the supernatants of 4-day-old cultures as an indicator of the secretory activity in the fungus. The activity values were higher, and reached earlier, in the cultures induced with farnesol (2.4 U/L) compared with the non-induced low inoculum density culture (1.5 U/L).

### Effect of inoculum density on farnesol production

The concentration of farnesol produced by *O. piceae* CECT 20416 was quantified in GPP cultures inoculated with 10^5^, 10^6^, and 5 × 10^6^ cells/mL, respectively. Farnesol was only detected in the last two cases (Table 1), reaching the maximum concentration (3 µM) at 16 h in the cultures with the largest inoculum size, and 1.8 µM after 24 h for the cultures inoculated with 10^6^ cells/mL. Farnesol concentration dropped to 0.8–1 µM after 40 h of culture.

**TABLE 1:** Farnesol concentration (µM) detected in the culture supernatants by HPLC-MS at different time points^*b*^

Inoculum (cells/mL)	Sampling time			
	8 h	16 h	24 h	40 h
10^5^	ND^*a*^	ND^*a*^	ND^*a*^	ND^*a*^
10^6^	0.57 ± 0.05	0.75 ± 0.22	1.86 ± 0.43	0.96 ± 0.07
5 × 10^6^	0.64 ± 0.08	3.03 ± 0.54	2.67 ± 0.32	0.83 ± 0.03

^*a*^ ND, not detected.

^*b*^ Measurements were performed in triplicate.

### Genomic analysis

The genome of *O. piceae* CECT 20416 was sequenced due to the biotechnological interest of this wood-staining fungus, which is also able of producing biofilms, secreting a versatile lipase, and forming consortia with bacteria. The draft genome sequence of *O. piceae* CECT 20416 was assembled into 46 contigs using *O. novo-ulmi (GCA_000317715.1*) as the reference genome, with N50 equal to 4.45, L50 of 3 and a total length of 32 Mb, GC content of 52.32% and gene count of 8,857. Among the predicted open reading frames, 8,479 (95.7%) had homologs in the nr/nt database, but only 3,007 (34.0%) and 6,593 (74.4%) could be assigned to identifiers from the Kyoto Encyclopedia of Genes and Genomes (KEGG) and Gene Ontology (GO) databases, respectively. Furthermore, 796 proteins were predicted to have signal peptides by SignalP 5.0. The search of enzymes active on carbohydrates in DbCan2 (https://bcb.unl.edu/dbCAN2/) predicted 311 CAZYmes with several relatively abundant families ─GH3, GT2, GH18, AA1, AA9, GH5, GH16, AA3 and GH76─, 149 of which were predicted to be secreted and 34 to be transmembrane. Enzymes active on chitin and involved in cell wall remodeling were found ─15 genes─, belonging to chitin synthases families GH18 and GT2. The set of oxidoreductases involved in lignocellulose degradation ─53 genes─ were mainly represented by families AA9 (10) and AA1 (9), associated with the oxidation of crystalline cellulose and diphenols, respectively, and family AA3 (9) formed by aril alcohol and glucose oxidases, and cellobiose dehydrogenase. These data were within average of organisms from the same genus (https://mycocosm.jgi.doe.gov/OphpiCECT20416_2/OphpiCECT20416_2.home.html).

### Molecular response to QS induction with farnesol

The molecular response to QS induction with farnesol in *O. piceae* was investigated under conditions expected to prevent crosstalk with other pathways involved in morphological transition. In brief, GPP medium was inoculated with 10^5^ cells/mL and grown without inducer addition (control group) or adding 100 µM farnesol 24 h after inoculation. Samples collected 22, 47, and 68 h post-farnesol induction were used to determine mycelium dry weight and to extract RNA for transcriptomic assays, as well as intracellular and extracellular proteins for proteomics.

The data from this experiment confirmed that fungal morphology was largely influenced by the QS signal. The higher proportion of hyphae in the farnesol-induced cultures was confirmed both from the dry weight of mycelium in each condition and by the higher yeast percentage in the controls, revealed by turbidimetry and observed by microscopy (Fig. S1). At 22 h post induction, the cultures were in the early exponential growth phase and at 47 h in the mid-exponential phase, reaching the late exponential phase at 68 h (Fig. S2A).

**Fig 2:**
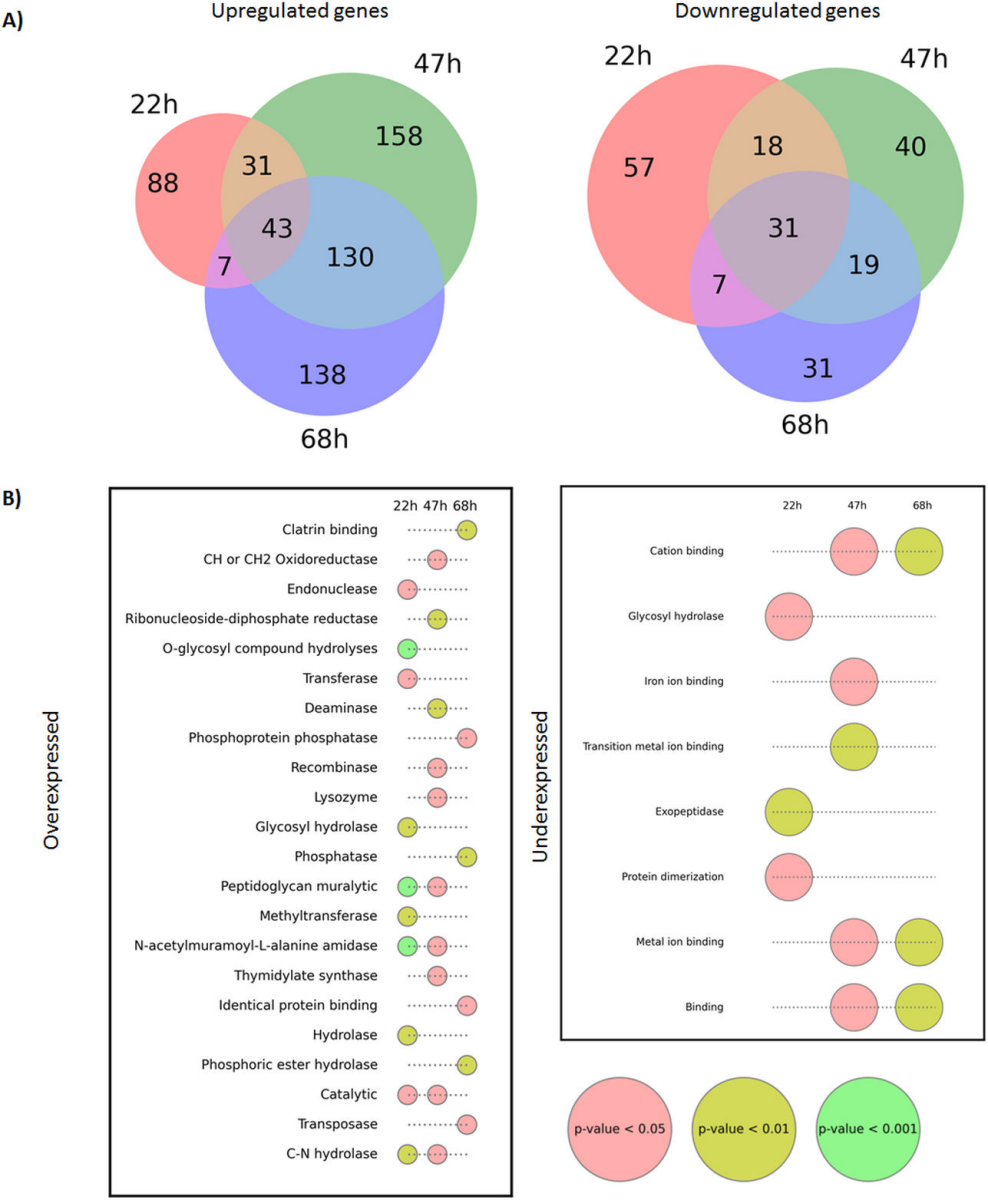
(A) Venn Diagrams of the number of genes found to be upregulated and downregulated after induction with farnesol. The criteria to consider a gene up- or downregulated were set at log_2_FC > 1.5 or log_2_FC < 1.5 and FDR *P*adj < 0.05. (B) Molecular functions ontologies (MF) significantly over-represented (*P*-value < 0.05) in the function enrichment analyses within the genes overexpressed (left panel) and the underexpressed (right panel). Gene sets are displayed as long as at least five genes belong to each ontology term. The dots represent an overrepresented MF for a given time point, and the color corresponds to the statistical significance of each ontology. A vacant circle represents that the MF is not over-represented for a given time point. (A) Venn Diagrams of the number of genes found to be upregulated and downregulated after induction with farnesol. The criteria to consider a gene up- or downregulated were set at log_2_FC > 1.5 or log_2_FC < 1.5 and FDR *P*adj < 0.05. (B) Molecular functions ontologies (MF) significantly over-represented (*P*­-value < 0.05) in the function enrichment analyses within the genes overexpressed (left panel) and the underexpressed (right panel). Gene sets are displayed as long as at least five genes belong to each ontology term. The dots represent an overrepresented MF for a given time point, and the color corresponds to the statistical significance of each ontology. A vacant circle represents that the MF is not over-represented for a given time point.

mRNA was sequenced in duplicates for control and farnesol-induced cultures, with an average count of 12.5 million reads per sample. 89.1% of the sequences were aligned to the reference genome, and 88.8% of them were unique and distinct alignments. Sample duplicates showed good correlation, clustering in a principal component analysis (PCA) (data not shown). Genes overexpressed (*P*adj < 0.05 and log_2_FC > 1.5) were more numerous at every timepoint than underexpressed genes (*P*adj < 0.05 and log_2_FC < −1.5). As shown in the Venn Diagram (Fig. 2A), 595 genes were found to be upregulated in cultures with farnesol at any time point, while 203 were downregulated. Although differences in transcription are apparent at 22 h, with 190 over- and 121 under-transcribed genes, major differences occurred at 47 h, with 362 genes upregulated and 108 repressed. Together with treatment-dependent responses, time dependency is also evident, given that only 43 genes were upregulated at the three sampling points.

Analysis of the GO enrichment data showed overrepresented molecular functions among both the upregulated and downregulated set of genes (Fig. 2B). Among the over-transcribed, regulatory elements were found at every timepoint, negative regulators of signaling and cell communication at the early and late timepoints, and genes related to the catabolism of carbohydrate derivatives and peptidoglycans, response to stimulus and defense in the earliest sampling time (22 h). Amid the under-transcribed set of genes (*P* < 0.01), the overrepresented functions were associated to biosynthesis of organonitrogen compound and exopeptidase activity at any sampling time, metal ion binding at 47 and 68 h, and nitrogen compound metabolism at 68 h (Fig. 2B).

### Interpretation of transcriptomic data using self-organized maps

Self-organized maps (SOMs) are an excellent tool to provide an intuitive global view of massive transcriptomics data, clustering genes according to their expression patterns and to their function. The SOMs obtained from the RNAseq data of the 18 samples analyzed are depicted in Fig. S3. Significant differences between treatments or timepoints were not observed for the genes with the lowest transcription levels, clustered in the top left corner of each SOMs. Furthermore, most of their products were not detected by proteomics. Similarly, no substantial variations were observed in the expression profile of most of the highly transcribed nodes, located in the upper right corner of the SOM plots. Slightly higher expression in induced samples can be observed in nodes 416 (at 22 and 47 h) and 419 (at 68 h), while it was reduced in nodes 357 and 378 at 47 h. However, the most relevant nodes, those displaying differential treatment-dependent transcription profiles across timepoints, cluster together at the upper center and center of SOM, node clusters vertex V409-V410-V411-V412-V389-V390 (C1) and V301-V302-V303-V290-V281-V282-V261-V262 (C2), which show higher levels of transcript under treatment, and clusters V48-V22-V1 (C3) and V20-V21-V42 (C4), which show the opposite behavior. Apart from these clear clusters, some other isolated nodes with differential transcription were detected.

Across C1, node V411 gets most of the attention, as the differential transcription of this node between treatments is over 64 times higher when treated with farnesol, with surrounding nodes mimicking this behavior, although in a less pronounced way. Proteomics confirmed that proteins from this node could not be detected in the untreated samples, while for the surrounding nodes, some proteins were found under all conditions. Cluster C1 displayed time-dependent transcription for some nodes, but with a clear upregulation under treatment: transcription levels were up to 16 times higher under farnesol treatment.

Clusters C3 and C4 displayed lower transcription levels under treatment and with some time-dependent differences. For example, nodes V1 and V48 only show a small treatment-dependent variation at 47 and 68 h while that difference is more than double at the initial time point.

### *Modeling of the adenylate cyclase of O. piceae* and *in silico docking of QSMs*

Adenylate cyclase plays a crucial role in the response to farnesol (4, 17), but the structural traits of Cyr1 from *O. piceae* are unknown. Considering that its amino acid sequence differs from that of *C. albicans*, the Cyr1 from *O. piceae* was modeled using the Swissprot server (Fig. S4). According to the model, the regulatory region contains a Rec receiver domain (Ras-associating domain), a GAF small molecule-binding domain (Leu-rich-repeat receptor), a HisK, histidine kinase domain (protein phosphatase), and a catalytic domain (Fig. S4A). *In silico* docking experiments of Cyr1 with farnesol and 3-oxo-C12-HSL (Fig. S4B and C) suggested that these QSMs interact with the catalytic domain.

## DISCUSSION

*Quorum sensing* is one of the main mechanisms used in nature to promote the coordinated response of microbial communities to certain stimuli. These processes have been studied in detail in prokaryotes, but among the fungal kingdom, most information refers to *C. albicans* due to its clinical relevance. The QS signals involved in the morphological transition in this microorganism and the most important signaling cascades that they activate are fairly well known (5, 27, 33), but little is known about what occurs in other fungi and yeasts. We are interested in the molecular components involved in the QS response to farnesol in *O. piceae* CECT 20416, and very especially in the transcriptional events related to yeast-to-hypha transition, which is essential for enhanced secretion of relevant enzymes and formation of biofilm (27, 29). *E,E*-farnesol promotes filamentation in this dimorphic fungus through QS mechanisms, enhancing the production of OPE, an extracellular versatile lipase, and the adhesion to solid surfaces (27) suggested that the transcriptional repressor Tup1 might mediate signal transduction, as well as the impact of the inoculum density on yeast or hyphal growth. Based on these findings, we undertook a study on the actors involved in the morphological transition in *O. piceae* and in the transcriptional regulation of the process.

The discussion that follows below involves a description of the main changes in gene expression/protein abundance that we detected by proteomics and transcriptomics. While some of these data are highly suggestive and consistent with previous work in *Candida albicans*, the conclusions should be buttressed by RT-PCR experiments to confirm changes in transcription of genes that we hypothesize are critical for the response to farnesol and by targeted deletion of a selected set of genes to confirm that they are physiologically involved in the QS response.

### Growth and morphology

The optimal culture conditions to study these events demonstrated that *O. piceae* CECT 20416 grows primarily as yeast when inoculated at low cell density in GPP, a minimal medium with glucose and proline as C and N sources, respectively (Fig. 1). Under these conditions, farnesol is not produced and accumulated and, therefore, there is no crosstalk between the QS molecule and other inducers of morphological transition. The influence of the nitrogen source on yeast-to-hyphae transition agrees with the induction observed using arginine, asparagine, or ammonium in other *Ophiostoma* species (31, 35). Regarding the inoculum, cultures inoculated with a low microbial density and later treated with farnesol produced more hyphae and extracellular esterase activity than untreated ones, mimicking the effect of inoculation with high cell densities, which agrees with previous results (27, 29). In addition, farnesol accumulated in the culture supernatants was only detectable in cultures inoculated with 10^6^ and 5 × 10^6^ cells/mL (Table 1). Apart from this, electron microscopy confirmed that the yeasts’ size was larger if the medium was inoculated with high cell densities (Fig. S1), which is reasonable since the yeast initiate the formation of the germ tube when transitioning to hyphae (30).

### Molecular response to induction of QS by farnesol

The effects of farnesol addition on gene transcription and expression in early, mid, and late stages of the exponential phase of cultures in GPP medium were studied by omics techniques. Up- or downregulated genes accounted for 9.4% of all predicted genes in the genome indicating a global response. To elucidate the farnesol signaling mechanism in *O. piceae,* we relied on the information described for *C. albicans* (36, 37) and searched for protein homologs. Most of the components of the HOG MAPK pathway were identified since kinases are highly conserved across genera. The enzymes involved in the Ras1-Cyr1/cAMP-PKA cascade are also conserved, except for Cyr1 adenylate cyclase (g6596) that in *O. piceae* is composed of 2,400 amino acids, 700 more than in *C. albicans*.

Given this large sequence difference, we built the model of the Cyr1 protein from *O. piceae* to compare its structure with that of *C. albicans* (38). Of the four domains identified (Fig. S4), the regulatory regions present the highest differences with *C. albicans* Cyr1, while the catalytic domain shows the highest sequence homology. In *C. albicans,* this domain can directly bind farnesol and other QS molecules such as the bacterial *n*-acyl-homoserine lactones, which control the catalytic activity (4). The 3D structural models of the catalytic domain from *O. piceae* suggest that it acts as a dimer, and molecular docking indicated that this dimer can bind farnesol and the bacterial QS molecule 3-oxo-C12-HSL (Fig S4B and C). As shown by other authors, a conformational change of Cyr1, depending on the signals, plausibly determines the activation or repression effect (4, 39, 40). Structural differences between Cyr1 in *C. albicans* and *O. piceae* might be the cause of the different mode of action of farnesol if both organisms.

On the other hand, the homology of the genes encoding the transcription factors from *C. albicans: cph1* (g7038), *cph2* (g6492), *tec1* (g6388), *efg1* (g1146), *czf1* (g4040), *rfg1* (g2405), and *nrg1* (g5887), which have a role in the farnesol signal transduction was studied, and the expression of the homologous genes in *O. piceae* CECT 20416 analyzed. Some of them showed time and treatment-dependent transcription differences; however, no significant differences were observed for *ras1* (g7886, g5725, g8145), *cyr1* (g6596), or protein kinases pka (g2527), tpk1 (g7253), tpk2 (g7928), *ste11* (g3465), *hst7* (g1934), or *cek1* (g8391). One hypothesis to explain why there are not significant changes in the expression of these genes is the regulation that may occur at the posttranslational level.

In *C. albicans*, farnesol inhibits Nrg1 degradation blocking the cAMP-PKA pathway, which, in turn, upregulates *tup1* that inhibits filamentation (41). In induced cultures of *O. piceae*, transcription of the *nrg1* repressor is downregulated while that of the *tup1* repressor remains constant. Furthermore, *efg1* and *czf1*, which promote transcription of hyphal-specific genes, are upregulated at 22 and 47 h, while in *C. albicans,* they are unaffected by farnesol. This may be sufficient to overcome the repressive effect of *rfg1* at every timepoint.

Finally, no farnesol import-export mechanisms have been described so far in fungi (4, 42, 43). In *O. piceae,* we have identified an ABC-2 type transporter (g7502) with high homology with several ABC transporters and more specifically with a human sterol transporter ABCG5/ABCG8 (44). The transcript coding for this protein is the highest overexpressed in farnesol cultures at all sampling times ─ log_2_FC greater than 8 for all time points ─ and the protein is also overrepresented in the proteome analysis. This makes g7502 a potential target to gain insight into the farnesol transport in fungi.

### Global metabolic changes

The addition of farnesol to *O. piceae* cultures is expected to result in changes that might affect central as well as secondary-metabolism pathways. Data analysis showed that deregulation of metabolism induced by farnesol is centered around three functional clusters: lipid, carbohydrate, and secondary metabolic pathways. Metabolic genes are ubiquitous in SOM, but they seem to be more prevalent in nodes upregulated under farnesol induction. The genes associated with lipid metabolism are mainly clustered around node V218, while those involved in carbohydrate catabolism are closer to V28 and V8.

With minor time-dependent differences, lipid metabolism is upregulated throughout the exponential phase of growth, especially at 22 h. Higher transcription levels were detected for oxoacyl carrier protein reductase (g3969 [V10]), malonyl-acyl carrier protein transacylase (g5427 [V165]), acyl-dihydroxyacetone phosphate reductase (g5161 [V76]), long chain fatty acyl-CoA ligase (g2484 [V218]), bifunctional p-450 NADPH-p-450 reductase (g5228 [V218]), 3-ketoacyl thiolase (g6916 [V174]), and a peroxisomal dehydrogenase-hydratase-epimerase (g8122 [V218]). Likewise, some genes that code for proteins involved in glycerol metabolism like glycerol-3-phosphatase (g3083 [V217]), dihydroxyacetone kinase (g218 [V217]), and phosphoglycerate mutase (g1308 [V29]) are also upregulated. In this sense, glycerol accumulation is related with specific responses to oxidative stress described for *C. albicans* (45).

Regarding carbohydrate metabolism, upregulation affects post-glycolytic enzymes such as aldehyde dehydrogenase (g8804 [V76]) and NADP-alcohol dehydrogenase (g656 [V28]) but also xylose dehydrogenase (g2503 [V28]), a hexose transporter (g6009), a transaldolase (g1772 [V8]) are upregulated.

As for secondary metabolism, melanin biosynthesis is the highest upregulated cluster. Given the likely protective activity of melanin, upregulation of these genes will be discussed further in section 3.7.

### Protection against oxidative stress

Farnesol is known to produce oxidative stress in *Candida* species (37, 46) and other fungi (36, 47). However, this effect is highly dependent on concentration and may induce ROS formation and apoptosis in *C. albicans* and other fungal pathogens, such as *Penicillium expansum* and *Aspergillus nidulans* (48, 49), while at low concentrations, farnesol activates the catalase-encoding gene (*cat1*) resulting in a ROS-protective effect (49, 50). In general, the origin of such an increase in ROS is not completely clear, as it might be attributed directly to farnesol or triggered by an increased metabolic rate (49).

Independently of the causative agent, a strong response to ROS has been observed in *O. piceae*. Glutathione and glutathione-dependent enzymes involved in the defense against oxidative stress and ROS degradation are differentially transcribed, the genes of glutathione *S*-transferases are ubiquitously upregulated (g3725 [V57], g3008 [V118]), and those of glutathione peroxidase (g493 [V110]) are upregulated during the early response. On the other hand, the degradation of superoxide anions requires their breakdown into hydrogen peroxide and oxygen (catalyzed by superoxide dismutase) before the intervention of peroxidase, in *O. piceae* the superoxide dismutase (g1708 [V379]) displayed transcriptional changes upon treatment, being heavily downregulated. This was corroborated by the proteomic analysis where the protein coded by g1708 is underrepresented at every sampling point in the farnesol-induced cultures. Contrarily, the peroxidase-catalase (g3481 [V39]), enzymes involved in aldehyde-detoxification dependent and independent on glutathione (g6535 [V8], g6352 [V31]) are upregulated in farnesol-induced cultures. All these findings suggest that farnesol does not promote the formation of superoxide anions, but it seems to increase production of other oxygen reactive species given the observed buffering response.

### Farnesol biosynthetic pathway

Since QS molecules are autoinducers (1), it is expected an upregulation of the farnesol biosynthetic pathway upon induction with farnesol. In most fungi, terpenoids are synthesized through the mevalonate pathway using acetyl-CoA as a precursor. In this case, the coding genes of enzymes involved in terpenoids synthesis, like diphosphomevalonate descarboxylase (g6105 [V281]), isopentenyl diphosphate isomerase (g2045 [V259]), and farnesyl pyrophosphate synthase (g3640 [V237]), mapped closely in SOM and did not display significant differences in their transcription patterns. However, the gene encoding the geranyl pyrophosphate synthetase (g1524 [V139]) is significantly upregulated for the two later time points. In the case of enzymes downstream the farnesyl pyrophosphate, as farnesyl transferases (g2414 [V323], g6269), squalene synthase (g1783 [V258]) and squalene monooxygenase (g2694 [V47]) we could not detect significant changes in gene expression. In this sense, it has been reported previously in *C. albicans* that transcription levels cannot account for treatment-dependent variations of farnesol biosynthesis (51)*,* suggesting that regulation is driven by the concentrations of the precursors that would likely be increased as a consequence of upregulating fatty acid and glycerol metabolism.

### Plasma membrane and cell wall remodeling

In the GO term analyses, it is shown that the components of membranes are enriched for all upregulated and repressed gene clusters at every time point (Fig. 2), highlighting the importance of this compartment. Cell-membrane variations were expected as morphological transition was already observable 22 h after inoculation. Together with cell membrane, cell wall was also expected to vary as a result of changes in ergosterol, chitin, β1–3 and β1–6 glucans biosynthesis (52).

Regarding enzymes active on polysaccharides, five genes were found coding for GT2 family enzymes, glycosyl transferases and chitin synthases, to be significantly upregulated at 47 h (g7552 [V165], g1153 [V75], g1052 [V15], g1236 [V77], g1235 [V76]), as well as five genes coding for enzymes of families GH76, GH16, GH72, GH76, and GT32 (g3926 [V78], g7551 [V138], g4683 [V78], g7637 [V197]). Downregulated gene coding for enzymes active on carbohydrates was not so common, only a GH16 (g3810[V379]), a glucan binding protein (g8168 [V399]), and an oxidase (g4409 [V106]) were undertranscribed for early timepoints, and a GH76 (g8566 [V410]) and arabinofuranosidase (g4864 [V21]) for later time points.

Morphological transition also needs of other changes related to transporters or anchor proteins. We found genes coding for adhesins to be upregulated for every time point with peak expression after 47 h (g4077 [V38], g6709 [V38], g3737 [V38]). These adhesins may be directly involved in biofilm formation, as farnesol has been previously reported to promote biofilms formation (29). Regarding the regulation of ABC transporters, some genes coding for these proteins are upregulated at the earliest time point (g5535 [V162], g7720 [V182]) in what appears to be a protective mechanism against farnesol, while others are upregulated in all sampling points (g7502 [V79], g499 [V29], g6797 [V27]). On the other hand, genes coding for proteins conferring drug resistance such as major facilitator superfamily transporters (MFS) (g7322 [V23], g6509 [V400], g4710 [V399], g3586 [V21]) or metal transporters (g1084 [V106], g2172 [V232], g7542 [V401], g4789 [V415], g7362) are mostly repressed in farnesol-treated cultures. The proteomics data confirmed this trait for the metal transporters with higher levels of transcripts, g1084 and g2172.

### Melanin biosynthesis upregulation

Melanin is a chemically diverse amorphous polymeric pigment that confers protection against environmental stresses such as ultraviolet and ionizing radiations, as well as against oxidizing agents. The genes identified in *O. piceae* encoding proteins involved in melanin biosynthesis were from the polyketide synthase pathway, the most common in Ascomycetes (53). It is especially relevant that all the genes involved in the biosynthetic process were upregulated, not only those mediating transformations of 1,3,6,8-tetrahydroxynaphthalene but also those responsible for precursor biosynthesis. Genes involved in naphthalene radical’s transformation: scytalone reductase (g627 [V10]), dihydroxynaftalene reductase (g3969 [V10]), and scytalone dehydratase (g3389 [V10]), coexpresed and mapped together in SOM node v10, were upregulated for every timepoint, and their gene products could only be detected in farnesol-induced cultures. Furthermore, several genes coding for upstream enzymes with the ability to shift equilibrium toward acetyl-CoA such as aldehyde dehydrogenase (g8804 [V76]), NADP-alcohol dehydrogenase (g656 [V28]) or long chain fatty acyl-CoA ligase (g2484 [V218]) showed upregulation compared to control cultures. The strong induction of this functional cluster in the early exponential phase (47 h) reveals the effect of farnesol on melanin biosynthesis at the transcriptional level.

All these results together, analyzing the molecular components of the QS system in *O. piceae* CECT 20416 suggest the main physiological adaptations caused by the presence of the autoinducer farnesol, such as cell wall remodeling, ROS protection, and melanin biosynthesis, as well as key genes involved in farnesol transport and signaling. These predictions can be useful for biotechnological applications and could be extrapolated to clinically relevant fungi phylogenetically related to Ophiostoma.

## MATERIALS AND METHODS

### Strain and growth conditions

*O. piceae* CECT 20416 was grown on solid potato-dextrose agar medium at 28°C for maintenance. Experiments were carried out in liquid minimal medium at 28°C and 180 rpm. The phosphate minimal medium contained glucose or sucrose as carbon source, and proline or arginine as nitrogen source. According to their components, the media were denominated GPP (with glucose and proline), SPP (sucrose and proline), GPR (glucose and arginine), and SPR (sucrose and arginine). The media contained 20 g/L glucose or sucrose, 1.15 g/L L-proline or L-arginine, 200 µg/L thiamine·HCl, 200 µg/L pyridoxine, 20 µg/L biotin, 100 µg/L CaCl·2H_2_O, 500 µg/L H_3_BO_3_, 400 µg/L ZnSO_4_·7H_2_O, 400 µg/L MnSO_4_·7H_2_O, 200 µg/L Na_2_MoO4·2H_2_O, 200 µg/L FeCl_3_·7H_2_O, 40 µg/L CuSO_4_·5H_2_O, 4 g/L H_2_PO_4_, 3.2 g/L Na_2_HPO_4_, and 0.5 g/L MgSO_4_·7H_2_O [derived from (54)].

First, 3-day-old pre-cultures in GPP medium were used to inoculate the different liquid media. Cultures were filtered through Miracloth (EMD Millipore) to remove hyphae, recovering the yeasts in the flow-through and centrifugating at 13,000 × *g* for 5 min. The pellet was washed (2×) and resuspended in 50 mM phosphate buffer. The number of yeasts per milliliter of inoculum was calculated using a Thoma counting chamber under the microscope. For the experiments, 18 biological replicates were used both for control cultures and for induction with farnesol. Groups of six biological replicates were pooled together to give three technical replicates that were individually analyzed. Erlenmeyer flasks (250 mL) with 50 mL of medium were inoculated with 10^5^ to 5 × 10^6^ cells/mL. In the treatments with farnesol, a final concentration of 100 µM of (*E,E*)-farnesol (Sigma 277541) was added to 24 h cultures. Flasks without added farnesol were used as controls. Samples were taken at different time points depending on the experiment.

### Morphology and growth evaluation

Culture morphology was determined counting yeast and hyphae cells under an Axioskop 2 microscope (Zeiss). As above, 18 biological replicates were used both for control cultures and for induction with farnesol. Groups of six biological replicates were pooled together to give three technical replicates that were individually analyzed. Three visual fields of the samples were examined with a 40× objective and the yeast/hypha ratio calculated (55). In selected samples, a more detailed study was carried out using scanning electron microscopy. In this case, the cultures were filtered through sterile Fluoropore filters (EMD Millipore, FGLP01300) and fixed in 2.5% (vol/vol) glutaraldehyde for 4 h at 4°C; then, the filters were dried by successive incubations in 30%, 50%, 70%, 80%, 90%, and 100% (vol/vol) ethanol for 10 min each and subjected to a CO_2_ critical point treatment. Finally, the samples were metalized with gold and analyzed in a JEOL 6400 JSM microscope with an accelerating voltage of 20 kV at the National Centre for Electronic Microscopy (CNME, Madrid, Spain).

The dry weight of the mycelium in 24 h cultures was calculated by drying the material retained at 55°C for 24 h in a pre-weighed Miracloth filter. The turbidity of the cultures after filtration (OD_600nm_) was evaluated in a Shimadzu UV-160 spectrophotometer.

### Esterase activity assay

As explained before, the three technical replicates obtained from 18 biological replicates were individually analyzed. Generic esterase and lipase activities were assayed spectrophotometrically by quantification of the *p*-nitrophenol (ε410 15,200 M^−1^ cm^−1^) release from *p*-nitrophenyl butyrate (*p*NPB) in 25 mM Tris-HCl buffer, pH 7.2 (27). One unit of activity was defined as the amount of enzyme hydrolyzing 1 mol of substrate per minute under these conditions. Protein concentration was determined by the method of Bradford (Bio-Rad protein assay) using serum albumin as a standard.

### Farnesol quantitation in liquid culture

The three technical replicates were individually analyzed. Farnesol concentration was quantified by extracting 5 mL of culture media supernatants with hexane:ethanol (9:1). The organic phase was evaporated and resuspended in 125 µL acetonitrile. The solution was injected into an HPLC (Thermo Finnigan Surveyor, Thermo Fischer) equipped with an RP18 Aquapore column (Life Technologies), applying a linear gradient of water-acetonitrile 0%–100% in 20 min. Farnesol was detected by MS (Thermoscientific LXQ, Thermo Fischer) and quantified using a calibration curve of pure *E,E*-farnesol.

### Genome sequencing and assembly

Fungal DNA was extracted from the pellet of 1 mL of a GPP liquid culture during exponential growth phase according to the manufacturer’s instructions (DNeasy plant minikit, Qiagen). DNA was quantified using a Nanodrop 2000 (Thermo Scientific) and sequenced by Novogene Co. Ltd in an Illumina HiSeq platform (150 bp paired-end) yielding a 100 × coverage. Reads were assembled using SOAP *de novo* 2.04 (56), and scaffolds below 500 bp were masked. AUGUSTUS server was used for gene identification and functional annotation against the GO, KEGG, EuKaryotic Orthologous Groups, and NR databases (57).

For the analyses of putatively secreted proteins, a list of proteins with signal peptides was selected using SignalP5.0 (58) with a 0.5 threshold. Proteins with more than one transmembrane motif according to TMHMM Server2.0 (59) were identified as transmembrane proteins and filtered out. DbCan2-meta server with parameters HMMER (*E*-value < 1e−15, coverage > 0.35), DIAMOND (*E*-value < 1e−102), and HotPep (frequency > 2.6, hits > 6) was used to analyze putative carbohydrate-active enzymes (CAZymes, Lombard et al., 2014). Those predicted by two or more tools were kept for further analyses. Manual analyses of certain genes were performed against NCBI nucleotide or *Candida albicans* genome databases (60). Conserved protein-motifs were analyzed using InterProScan (61). Given the special interest in secreted and carbohydrate-active enzymes, Secretool and dbCan2 servers were, respectively, used for further annotation (62, 63).

### RNA preparation and sequencing

Eighteen biological replicates (samples of 1 mL) were taken from the control and farnesol-treated liquid cultures in GPP medium at 22, 47, and 68 h after induction. Nine samples were pooled to get two replicates of each treatment. Samples were centrifuged at 13,000 *× g* for 5 min, supernatant discarded, and pellets stored at −80°C until processed. RNA was extracted using a RNeasy plant minikit (Qiagen) following the manufacturer’s instructions, followed by a DNase treatment with TURBO DNA-free (Thermo Scientific). RNA quality and quantity were determined using Experion (Bio-rad) on-chip electrophoresis, where all samples met a RIN threshold >8. RNAseq was performed in an Illumina NovaSeq 6000 platform by Novogene Co. Ltd, yielding 150 bp-long paired end reads in FASTQ format.

### RNAseq processing and analyses

FASTQ read files were processed with Cutadapt (64), and quality control was performed using MultiQC (65). RNA reads were aligned to the genome of *O. piceae* CECT 20496 using STAR (66) and the nf-core RNAseq pipeline (67) allowing for unique mapping. Raw read counts were used for differential expression analyses using DESeq2 (68). The normalized log2 transformed gene read count showed similar mean and distribution patterns for all libraries and the PCAs clustered replicates together. Gene expression data were normalized and used to calculate log_2_ fold differences and adjusted *P*-values for every time point using treatment as the experimental factor (treatment with farnesol vs control). A Bonferroni-correction adjusted *P*-value < 0.05 and a log_2_ FC > 1.5 were set as thresholds to consider differences significant. Gene enrichment analyses were performed in R using the top GO package after creating a custom GO database from the genome annotations (69). Furthermore, data were visualized with the Venn Diagram and pheatmap R packages in combination with DESeq2 (70, 71).

To cluster genes according to their expression patterns and to their function, SOMs were trained (72). The number of nodes for the SOM model was set to 420 according to the size of the fungal genome (73). The R package Kohonen was used to train the model with the means of the rlog transformed gene counts as input for each time and treatment, alpha was set to decrease linearly from 0.05 to 0.01, and the number of iterations was set to 10,000 (68, 74). Learning rate and distance reductions were evaluated and deemed appropriate.

### Proteomic analysis

As described for RNA extraction, 1 mL of each culture was centrifuged at 13,000 × *g* for 5 min. The supernatant was concentrated in a centrifuge filtration system Centricon-Plus 3 kDa (Merck-Millipore) to a volume of 50 µL to perform secretomic analyses. For intracellular proteomics, cell pellets were freeze-dried and disrupted by grinding with a mortar and pestle. Five-hundred milligrams of ground biomass was resuspended in 1 mL of Tris-glycine buffer (0.025 M Tris–0.192 M Gly) plus 0.1% SDS and centrifuged at 12,500 × *g* for 40 min at 4°C, collecting the supernatant. The protein mixtures from the supernatant and those extracted from the pellets were analyzed in a nano-HPLC Easy-LC column (Proxeon) connected in series with a LTQ Orbitrap Velos analyser (Thermo Scientific). The gradient for liquid chromatography was linear, ranging from 0% to 35% of buffer B (0.1% of formic acid in acetonitrile) in A (0.1% formic acid and 2% acetonitrile in water) during the first 120 min and up to 20% for 20 more minutes. Proteins were identified querying MS/MS data against the *O. piceae* CECT 20416 annotated protein database using Sequest and Mascot. Identified peptides were validated with Percolator, and only those with a high confidence level were accepted (75). Predicted proteins were mapped to their respective gene SOM nodes to compare between observed transcription and translation levels although protein visualization only enables for qualitative analyses.

### Protein modeling

The predicted sequences that encoded relevant proteins were manually curated and subjected to three-dimensional modeling with the programs implemented by the automated protein homology-modeling server SWISS-MODEL [https://swissmodel.expasy.org/; (76)]. The models were comprehensively analyzed using PyMol 1.1 (http://pymol.org/) (77). Due to its length and heterogeneity, the model was built in four parts using four different crystal structures as templates: the RA domain of FLJ10324 from *Homo sapiens* (PDB 3EC8) from residue 300 to 400 (QMEAN −2.74); the flg22 transferase receptor from *Arabidopsis thaliana* (PDB 4MN8) from residue 400 to 990 (QMEAN −6.82); the PP2Ca-D38A metal binding protein from *Homo sapiens* (PDB 4RAF) from residue 990 to 1,300 (QMEAN −3.58); and the adenylate cyclase from *Trypanosoma brucei* (PDB 1F × 2) from residue 1,300 to 1,690 (QMEAN −4.93). The docking of farnesol and 3-oxo-C12-HSL in the model was performed using the automated SWISS-DOCK server (http://www.swissdock.ch/) selecting both ligands from the database.

## Data Availability

The genome sequence from *O. piceae* CECT 20416 v2 was deposited in the NCBI database under the accession number PRJNA1019113. Raw reads obtained from each replicate of the *O. piceae* CECT 20416 transcriptome growing in GPP with or without farnesol were deposited in the NCBI database under the accession number PRJNA906718. The processed data was deposited in the GEO database under the accession number GSE243253.

## SUPPLEMENTAL MATERIAL

Supplemental figures spectrum.00290-23-s0001.pdf Figures S1-S4.

ASM does not own the copyrights to Supplemental Material that may be linked to, or accessed through, an article. The authors have granted ASM a non-exclusive, world-wide license to publish the Supplemental Material files. Please contact the corresponding author directly for reuse.
