# Supplementary material for: Deciphering the molecular components of the quorum sensing system in the fungus Ophiostoma piceae
Source: Microbiol Spectr. 2023 Oct 5;11(6):e00290-23. doi: 10.1128/spectrum.00290-23 (PMC10715110; doi:10.1128/spectrum.00290-23)
Supplement: Supplemental figures — Figures S1-S4. [file spectrum.00290-23-s0001.pdf]

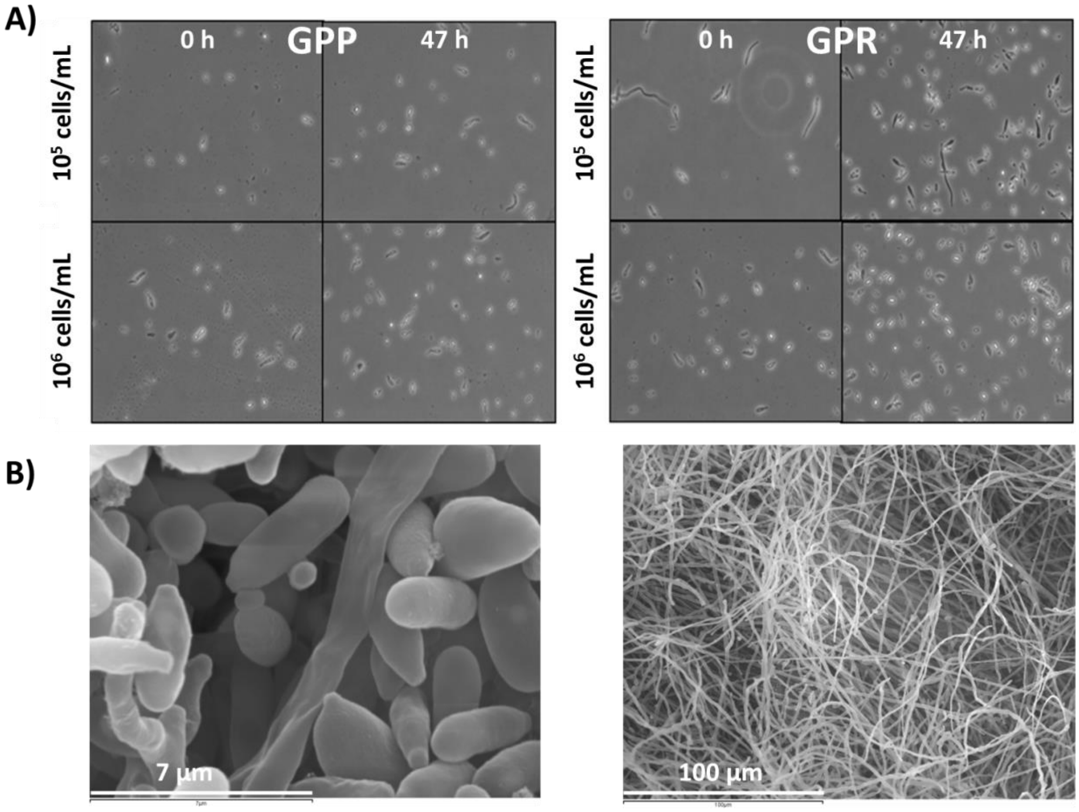

Figure S1. A) Light microscope (40x) images showing the morphology of cultures at 0 h and of 47 h post-inoculation in phosphate medium with glucose (G) or sucrose (S) as the carbon source, and proline (P) or arginine (R) as the nitrogen source. The media GPP (glucose-phosphate-proline) and GPR (glucose-phosphate-arginine) were inoculated at low (105 cells/mL) and high cell density (106 cells/mL). B) Scanning electron microscope (SEM) images of a control culture in medium GPP (left panel) and a culture induced with 100 um farnesol (right panel).

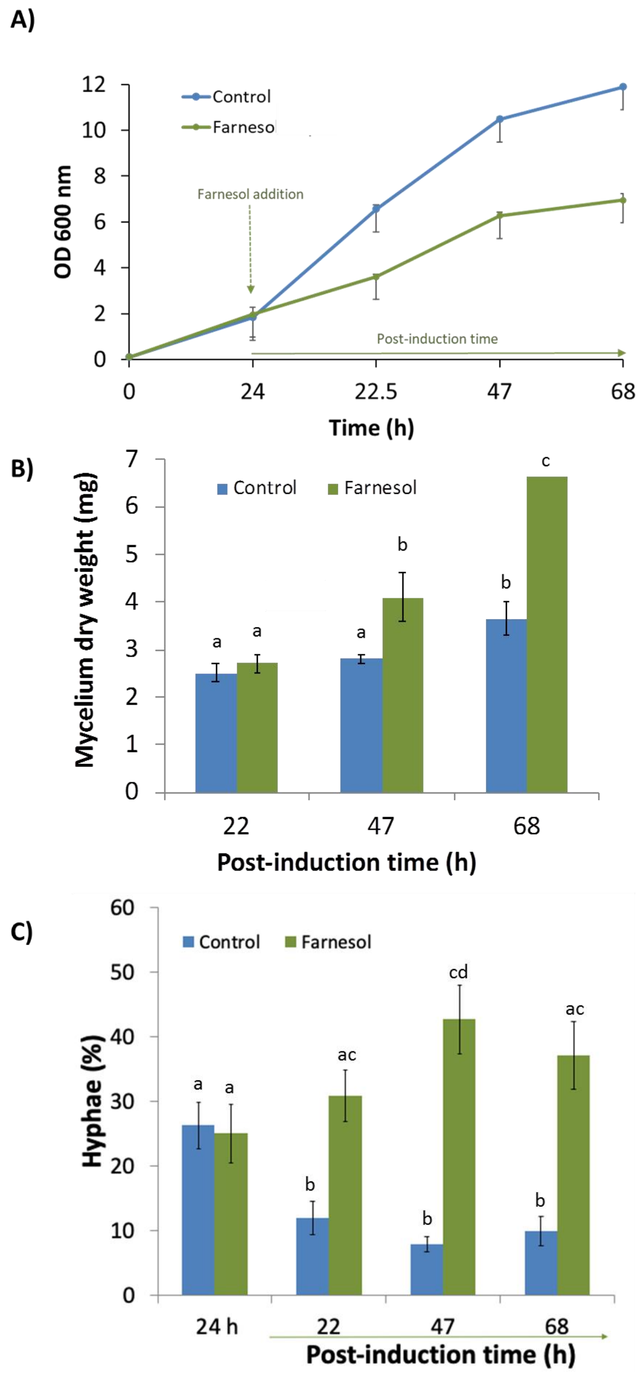

Figure S2. Morphology of control cultures of O. piceae CECT 20416 or induced with 100 uM farnesol. A) OD of the culture filtrates through Miracloth. B) Mycelium dry weight. C) Percentage of hyphae observed by light microscopy. Eighteen biological replicates were used both for control samples and for the induction with farnesol. Groups of six biological replicates were pooled together to give three technical replicates, that were individually analyzed. Error bars: standard error. Statistical significance of the results

was evaluated by ANOVA and Tukey tests.

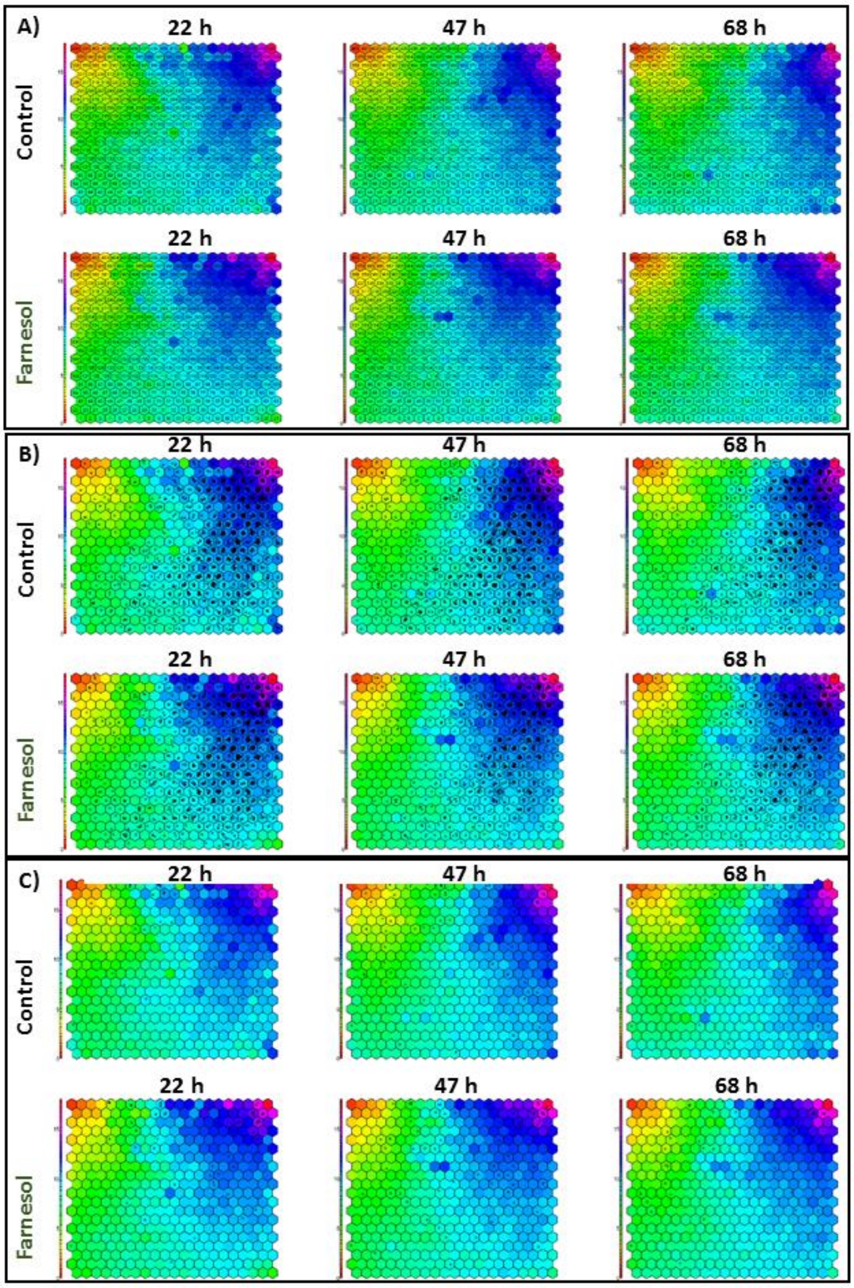

Figure S3. Self-organized maps (SOM) for comparative transcriptomic and proteomic analysis of control *vs.* farnesol-induced cultures. A) Transcriptomic data and numbering of the nodes. Each plot represents gene transcription levels for a given timepoint and condition. Each SOM node groups genes with similar expression patterns and the color gradient represents their transcription level. B) Location in SOM nodes of the proteins detected in the secretome in each condition. C) Location in SOM nodes of the proteins detected in the intracellular proteome in each condition

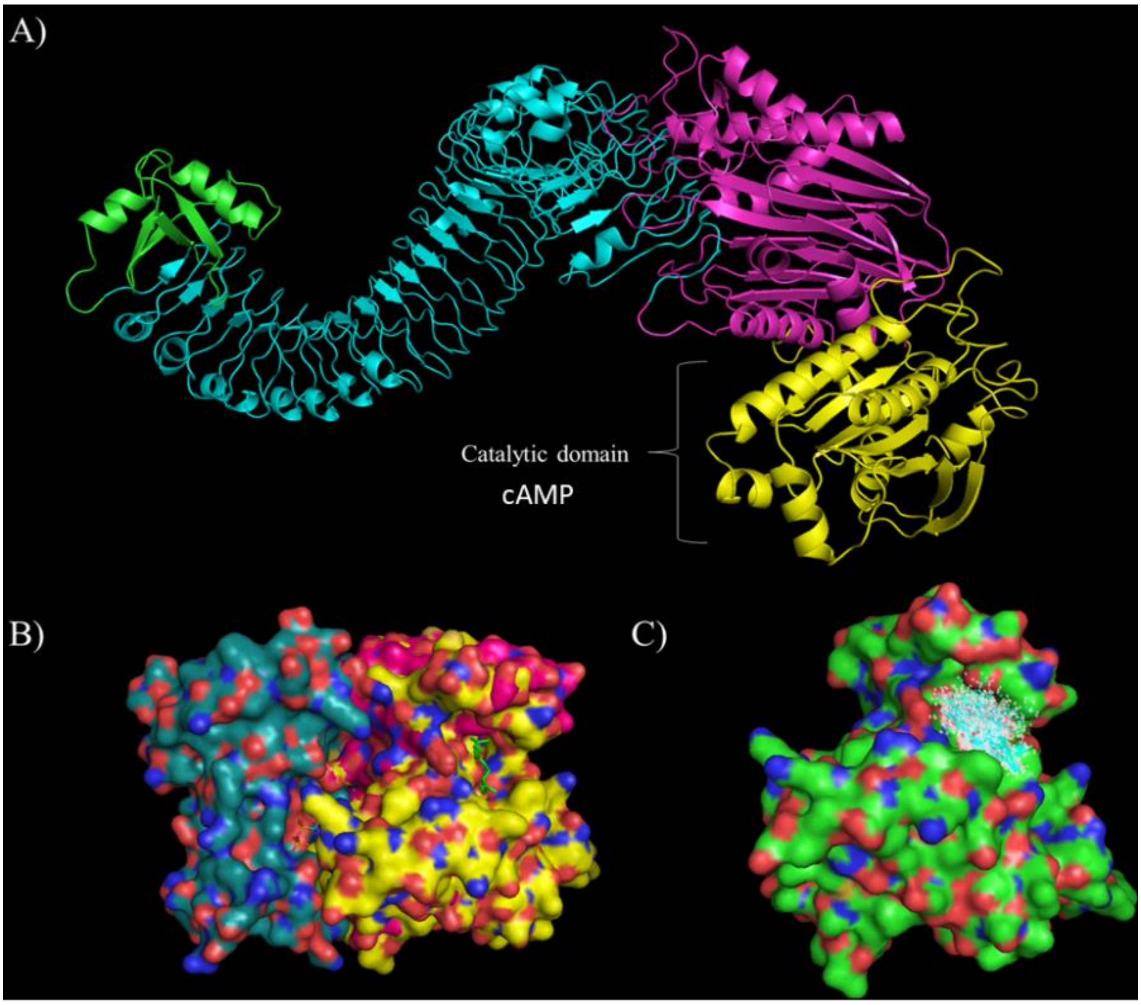

Figure S4. Modelling of the structure of the key integrator Cyr1 (adenylate cyclase). A) whole structure of Cyr1. B) Detail of the dimeric catalytic centre, showing the location of a molecule of farnesol (green). C) Monomeric unit of the catalytic centre with the potential binding sites of a molecule of 3-oxododecanoyl-L-homoserine lactone (light blue).
